# A Retrospective Study of Viral Molecular Prevalences in Cats in Southern Italy (Campania Region)

**DOI:** 10.3390/v14112583

**Published:** 2022-11-21

**Authors:** Maria Grazia Amoroso, Francesco Serra, Gianluca Miletti, Lorena Cardillo, Claudio de Martinis, Luisa Marati, Flora Alfano, Gianmarco Ferrara, Ugo Pagnini, Esterina De Carlo, Giovanna Fusco, Serena Montagnaro

**Affiliations:** 1 Department of Animal Health—Istituto Zooprofilattico Sperimentale del Mezzogiorno, Via Salute n. 2, 80055 Naples, Italy; 2 Department of Veterinary Medicine and Animal Productions, University of Naples “Federico II”, Via Delpino 1, 80137 Naples, Italy

**Keywords:** feline coronavirus, feline leukemia virus, feline panleukopenia virus, real-time PCR, co-infection

## Abstract

From 2019 to 2021, a retrospective molecular study was conducted in the Campania region (southern Italy) to determine the prevalence of viral diseases in domestic cats. A total of 328 dead animals were analyzed by Real-Time PCR for the presence of feline panleukopenia virus (FPV), feline leukemia virus (FeLV), feline enteric coronavirus (FCoV), rotavirus (RVA), feline herpesvirus type 1 (FHV-1), and feline calicivirus (FCV). The possible presence of SARS-CoV-2 was also investigated by Real-Time PCR. The cats included in this study were specifically sourced and referred by local veterinarians and local authorities to the Zooprofilactic Experimental Institute of Southern Italy (IZSM) for pathological evaluation. The samples consisted of owners, catteries, and stray cats. Results revealed: 73.5% positive cats for FPV (189/257), 23.6% for FeLV (21/89), 21.5% for FCoV (56/266), 11.4% for RVA (16/140), 9.05% for FeHV-1 (21/232), and 7.04 for FCV (15/213). In contrast, SARS-CoV-2 was never detected. FPV was more prevalent in winter (*p* = 0.0027). FCoV FHV-1, FCV, and RVA predominated in autumn, whereas FeLV predominated in summer. As expected, viral infections were found more frequently in outdoor and shelter cats than in indoor ones, although no statistical association was found between animal lifestyle and viral presence. The study showed a high prevalence of FPV, FeLV, and FCoV and a moderate prevalence of RVA, FHV-1, and FCV. Moreover, the prevalence of these pathogens varied among the cat populations investigated.

## 1. Introduction

Cats are very popular pets and have become an integral part of people’s lives. According to the “Centro Studi Investimenti Sociali” (CENSIS, www.censis.it), the number of cats in Italy is increasing every year. About seven million cats are registered in the official cat registry, but their number is certainly even greater if we consider the unregistered and stray cats. Since these pets play an important role in our society, viral infectious diseases affecting their health are of great interest.

The most common viral pathogens infecting cats are feline panleukopenia virus (FPV), feline leukemia virus (FeLV), feline enteric coronavirus (FCoV), rotavirus (RVA), feline herpesvirus type 1 (FHV-1), and feline calicivirus (FCV).

Feline herpesvirus type 1 (FHV-1) and feline calicivirus (FCV) are the most important causative agents of upper respiratory tract disease in cats, with rhinotracheitis, conjunctivitis, stomatitis, gingivitis, and nasal/facial ulceration being the most common clinical manifestations [1]. FHV-1 is a double-stranded DNA virus that belongs to the family *Herpesviridae* [2]. It is mainly transmitted by direct contact, contamination, and droplets [3]. FCV, a plus-stranded, non-segmented RNA genome virus belonging to the family *Caliciviridae* [4], is transmitted from sick and/or apparently healthy carrier cats to healthy cats via the nasal, oral, or conjunctival route and mainly by close contact. Although disease signs are generally mild, highly virulent viral variants can also cause very severe disease [5].

Feline panleukopenia is a highly transmissible viral disease caused primarily by Feline Panleukopenia Virus (FPV), but in rare cases, also by Canine Parvovirus (CPV) [6]. FPV, a virus belonging to the family *Parvovirinae,* is characterized by a linear, single-stranded DNA genome [7]. Feline panleukopenia is a highly morbid and fatal disease for all members of the family *Felidae* [8,9]. The disease can be prevented by vaccination, which is strongly recommended because FPV can cause severe diseases in cats [6]. Transmission occurs by direct (or fecal) or indirect contact, as the virus persists in a contaminated environment for long periods of time. Clinical signs include diarrhea with severe dehydration, lethargy, fever, anorexia, and immunosuppression [10].

Feline leukemia virus (FeLV) is an enveloped single-stranded RNA virus of the *Retroviridae* family infecting feline species. The viral disease is transmitted by “friendly” behaviors such as mutual grooming and sharing water and food. These are considered the main routes of infection, but the virus can also be transmitted through fights and bites [11]. FeLV is mainly associated with anemia, leukemia, and lymphoma in infected cats [12].

Feline coronavirus (FCoV) is an enveloped single-stranded RNA virus belonging to the family *Coronaviridae*. It causes an infection with high morbidity very common in cats, especially those kept in groups or at high densities, such as in kennels, shelters, and multi-cat households [13]. FCoV usually causes intestinal disease, but a biotype known as feline infectious peritonitis virus (FIPV) is the causative agent of feline infectious peritonitis (FIP), a fatal, immune-mediated disease. Following fecal–oral infection, FCoV replicates in enterocytes, particularly in the small intestine, and is shed in feces [14,15]. Usually the infected cats recover fully, and the virus is no longer shed. However, some animals become chronic carriers of the virus, which is continuously excreted in the feces [13].

Rotavirus, a species of the *Reoviridae* family, is an important pathogen that causes acute diarrhea in young animals of many species, including humans. Rotaviruses include 12 species (A–L), and to date, species A (RVA) is the most commonly detected in cats [16]. For these animals, RVA plays a minor role in clinical disease, and there is no routine screening for diarrheal manifestations in veterinary studies of small animals [17]. However, as pets, cats have intense contact with their owners and share the same environment with them. Therefore, they can be a source of pathogens that can infect humans, including rotavirus [16].

Given the lack of epidemiological studies on infectious diseases and viral agents in cats in Italy, especially in southern Italy, the aim of our work was to investigate the prevalence of FHV-1, FCV, FPV, FeLV, FCoV, and RVA in cats in the Campania region (Southern Italy). The study was performed by Real-Time PCR using protocols specific for the tested virus. Since cats are among the animals susceptible to COVID-19 infection [18], the owner cats referred from September 2020 were also tested for the presence of SARS-CoV-2.

## 2. Materials and Methods

### 2.1. Ethical Approval and Study Design

Ethical approval was not required for our study because no live animals were used in this investigation. Cats included in this study were referred by local veterinarians and local authorities to the Zooprofilactic Experimental Institute of Southern Italy (IZSM), for pathological examinations with the final aim to ascertain the causes of death. A total of 328 dead cats were sent to the IZSM over a 3-year period (2019–2021). Samples consisted of owners, catteries, and stray cats. Registration forms were collected to obtain information on the animals’ history. Only data from the complete history form were collected and considered as variables for risk analysis (year of death, season, location, and lifestyle). Data on gender, age, and breed were not analyzed because they were incomplete.

### 2.2. Materials

From 2019 to 2021, we investigated a total of 328 cat death cases. The carcasses were dissected by staff (veterinarians and laboratory technicians) in a necropsy room. The organs (spleen, brain, heart, lung, liver, spleen, and intestine) were removed with sterile scalpels, dissected, divided into sterile tubes, and sent to the laboratories of our institute for virological studies. In addition, rectal and oropharyngeal swabs for detection of SARS-CoV-2 were also collected from the owners’ cats starting in September 2020. Samples were analyzed by Real-Time PCR to detect FHV-1, FeLV, and FPV (viruses characterized by a DNA genome) and by Real-Time RT-PCR to detect viruses with an RNA genome: FCoV, FCV, RVA, and SARS-CoV-2. Viral analyses were carried out following veterinarians’ indications in accord to the anamnesis information they provided together with samples. Not all the 328 cats were therefore tested for all the 6 viruses, and not all the organs of the single animal were tested for the same pathogen.

### 2.3. Viral Nucleic Acids Extraction Procedures

Samples (25 mg tissue in 1 mL phosphate-buffered saline) from each organ were homogenized using a 4.8-mm stainless steel bead in a tissue lyser (Qiagen, Hilden, Germany) and then clarified by centrifugation at 1740× *g* for 5 min. Prior to extraction, all samples were artificially contaminated with 10 µL of murine norovirus (10^7^ PFU/mL), used as a quality control for nucleic acid extraction [19]. For specific detection of SARS-CoV-2, oro-pharyngeal and fecal swabs were pre-treated as indicated by Dakroub et al., 2022 [20]. Prior to extraction, 5 µL of an internal control (included in the TaqPath^TM^ COVID -19 RT-PCR kit) was added to the samples to check the quality of the extraction. The negative process control (NPC) was prepared using 200 µL of PBS instead of the sample. Nucleic acid extraction was performed from 200 μL of each homogenate or swab supernatant using the Qiasimphony automated extraction system (QIAGEN, Hilden, Germany) with the DSP Virus/Pathogen Mini Kit (Qiagen, Hilden, Germany; catalogue number (CN): 937036) according to the manufacturer’s instructions. Eluates (80 μL) were stored at −80 °C or processed immediately. The presence of PCR inhibitors was determined for each sample by monitoring the nucleic acid extraction control results in accordance with Amoroso et al. 2021 [21].

### 2.4. Real-Time PCR for the Detection and Quantification of Viral Genome

Real-time virus detection was performed in a final reaction volume of 25 μL with 5 μL nucleic acid extract. FHV-1, FeLV, and FPV viral genomes were analyzed by Real-Time polymerase chain reaction using the Quantitect Real-Time PCR detection kit (Qiagen, Hilden, Germany; CN: 204443). The presence of FCoV, FCV, and RVA genomic sequences was instead examined by Real-Time RT-PCR using the AgPath-ID™ one-Step RT-PCR kit (Thermo Fisher Scientific, Waltham, MA, USA). All reactions were carried out in single runs using primers (TemaRicerca-Castenaso, Bologna, Italy) and probes (Thermo Fisher Scientific, Waltham, MA, USA) specific for the virus tested (see Table 1) and following the thermal profiles indicated in the literature (Table 1). SARS-CoV-2 presence was investigated using the TaqPathTM COVID -19 RT-PCR kit (Thermo Fisher Scientific, Waltham, MA, USA). This involves binding probes to three target sequences specific for SARS-CoV-2. Each target is located between unique forward and reverse primers for the following genes: ORF1ab, N protein, and S protein. The reaction was performed according to the manufacturer’s instructions. All reactions were run on a Quant studio 5 System (Thermo Fisher Scientific Waltham, MA, USA). The following positive controls (kindly given by Friedrich-Loeffler-Institut) were employed: FCV strain F-9, FPV strain Lepzig 1163/6, and RVA strain Hrr 1/79. As FVH-1 positive control, the Tricat vaccine (Nobivac) containing the live attenuated strain G2620A was used. FCoV positive control consisted instead of a field strain kindly given by University of Bari (Italy). As FelV positive control, the ATCC strain FL-237 was employed. SARS-CoV-2 positive control was included in the kit. Each biomolecular assay was individually validated prior to being used for the viral screenings. In detail, robustness was monitored by adopting ad hoc amplification controls, as previously indicated [22]. Sensitivity and specificity were instead calculated according to Trevethan et al., 2017 [23] by analyzing 5 positive and 5 negative samples in two different days and involving two technicians. Both resulted at 100% for all the protocols carried out. Real-time specificity was instead assessed for each protocol by amplifying nucleic acids extracted from the other viruses under analysis. No cross-reaction among the viruses was observed, confirming the high specificity of the Real-Time PCR protocol adopted. 

### 2.5. Statistical Analysis

Data analysis was performed with MedCalc Statistical Software version 16.4.3 (Med-Calc Software, Ostend, Belgium; www.medcalc.org; 2016). Chi-square tests were used to compare proportions of positivity in relation to categorical dependent variables and to determine statistical significance within each class (year, season, location, and lifestyle). Variables associated with molecular prevalence of FHV-1, FCV, FPV, FeLV, FCoV, and RVA were analyzed in binary logistic models using JMP Pro version 15.0.0 (SAS Institute Inc., Campus Drive, Cary, NC, USA). *p* < 0.05 was considered significant. Significant differences between categories were quantified by calculating odds ratios (OR) and their 95% confidence intervals (CI).

## 3. Results

### 3.1. Viral Prevalence of Infection and Risk Factor Analysis

During the study period, 328 deceased cats were sent to IZSM as part of the National Sanitary System Service for diagnostic testing. Information on year, season, location, and lifestyle was recorded for all. Not all 328 cats were tested for all the 6 viruses because viral genome testing was performed following veterinarians’ indications. Therefore, at their request, 232 cats were tested for FHV-1, 213 for FCV, 257 for FPV, 89 for FeLV, 266 for FCoV, 140 for RVA, and 52 for SARS-CoV-2. For statistical analysis, an animal with viral DNA/RNA in at least one organ was considered positive for the disease. The overall results showed that 231/328 animals (70.4%) were positive for at least one pathogen, 57/328 (17.4%) showed the simultaneous presence of two viruses, 7/328 (2.1%) were positive for 3 viruses, while only three samples (3/328, 0.9%) tested positive for 4 pathogens (Table 2). The presence of two or more viruses accounts for about 1/3 (67 / 231.29.00%) of the total infections found.

FPV proved to be the most common viral pathogen with a prevalence value of 73.5% (189/257, 95% CI: 68.2–78.9%) (Table 3). FPV prevalence values observed in 2021 were statistically significant (*p*-value < 0.0001) than those observed in other years (Table 3). Univariate and multivariate analyses showed a statistical association between season (*p*-value = 0.0027) and molecular FPV prevalence. Indeed, the risk of FPV positivity was significantly correlated with winter (95.5% 95% CI: 89.5–100.0) with odds ratios of 10.05, 12.57, and 9.48 compared to spring, summer, and autumn, respectively (Table 3). Data analysis showed that there was no statistical association between FPV infection, location (*p*-value = 0.1798), and lifestyle (*p*-value = 0.57). However, the highest percentage of positive animals was observed in cats in the province of Naples (79.1%, 102/129, 95% CI: 72.1–87.0) and in outdoor cats (76.7%, 89/116, 95% CI: 69.0–84.4) (Table 3).

The FeLV genome was detected in 23.6% (21/89, 95% CI: 14.7–32.4) of the cats examined. FeLV positivity was 33.3% (95% CI: 19.08–47.6) in 2019, and this value was statistically significant (*p*-value = 0.0102) compared to the values observed in the other years (Table 4). A statistical correlation was also observed between FeLV molecular positivity and location. The risk of FeLV positivity correlated with the province of Naples (37.5%, 15/40, 95% CI: 22.5–52.5) with odds ratios of 4.6 and 4.5 for the provinces of Avellino and Benevento, respectively; no positive cases were detected in the provinces of Caserta and Salerno. In FeLV positive cats, no statistical significance was found in relation to season and lifestyle, although the highest prevalence value was found in animals in summer (32.1%, 95% CI: 19.5–44.6) and in cats living in shelters (32.0% 95% CI: 19.07–44.9) (Table 4).

FCoV was detected in 21.1% (56/266, 95% CI: 16.2–25.9) of the samples analyzed. Data analyses showed that there was a statistically significant difference in prevalence values with respect to year (*p*-value = 0.0015), and 2019 was the year with the highest prevalence (34.2%, 26/76, 95% CI: 23.5–44-8), followed by 2021 and 2020. Compared to the reference year 2021, the odds ratios for 2020 and 2019 were 1.9 (95% CI: 0.82–4.25) and 0.45 (95% CI: 0.23–0.89), respectively (Table 5). Of the 140 cats tested for RVA, 11.43% were positive to Real-Time RT-PCR. Data analysis showed that there was a statistically significant difference in prevalence values only with respect to year (*p*-value = 0.0001), with a prevalence value of 32.3% (10/31, 95% CI: 15.8–48.7) and an odds ratio of 0.16 (95% CI: 0.05–0.54) compared to 2021 (versus reference category). Results showed that there was instead no statistically significant difference in prevalence values with respect to location (*p*-value = 0.2896), although the highest percentage of positive cats was found in the province of Salerno (22.2%, 95% CI: 0.00–49.4), followed by Caserta (18.5%, 95% CI: 3.87–33.2), Napoli (11.6%, 95% CI: 4.04–19.15), and Avellino (3.4%, 95% CI: 0–13.2) (Table 6).

Regarding RVA, the results indicated no significant difference in viral molecular prevalence in different seasons (*p*-value = 0.573) and for different breeds (*p* = 0.7951), although the highest positivity was found in winter (16.7%, 95% CI: 1.76–31.6) and in outdoor cats (12.5%, IC 95% 4.86–20.14) (Table 6).

Low prevalence was detected for FHV-1 and FCV in the samples tested, with a percentage of positivity of 9.05% (21/232, 95% CI: 5.36–12.74) and 7.0% (15/213, 95% CI: 3.61–10.5), respectively. FHV-1 DNA detection was statistically correlated with year (*p*-value = 0.0323) and location (*p*-value= <0.0001). In particular, the year 2019 (16.9%, 9/53, 95% CI: 6.87–27.09, OR 0.21) and the province of Caserta (13.3%, 4/30, 95% CI: 1.2–25.5) were negatively associated with infection compared to the reference category. A similar scenario was found for FCV infections. However, in this case, the year with the highest prevalence value was 2020 (13.6%, 11/81, 95% CI: 6.12–21.0) and the province with the highest number of positive cats was Salerno (33.3%, 2/6, 95% CI: 0.00–71-7) (Table 7 and Table 8).

No SARS-CoV-2 RNA was detected in the animals studied, either in rectal or oro-pharyngeal swabs.

### 3.2. Viral Prevalence in the Different Organs Analysed

The frequency of detection of the viral genomes in the different organs studied is shown in Table 9. Briefly, FPV was the only pathogen found in all organs studied. Specifically, its genome was found in 61.4% (95% CI: 46.9–75.7) of brains, 70.8% (95% CI 62.1–79.6) of hearts, 68.2% (95% CI 61.6–74.7) of lungs, 70.2% (95% CI: 62.4–78.1) of livers, 77.5% (95% CI: 62.9–81.1) of spleens, and 71.5% (95% CI: 65.7–77.2) of intestines. Of the 34 heart samples analyzed, 41.4% (95% CI: 27.4–60.8) tested positive for FeLV and the viral genome was found in 11.1% (95% CI: 0.8–21.3), 11.5% (95% CI: 0.0–23.8), and 20.0% (95% CI: 4.3–35.6) of lungs, livers, and intestines, respectively. FCoV was found in all organs except brains, with positivity rates of 28.9% (95% CI: 14.5–43.3) in heart, 8.8% (95% CI: 1.9–20.7) in lungs, 16.3% (95% CI: 3.2–29.1) in liver, 15.8% (95% CI: 0.0–32.2) in spleen, and 18.1% (95% CI: 13.1–23.1) in intestine. The RVA genome was identified only in the liver (3.85%, 95% CI: 0.0–11.2) and in the intestine (10.4%, 95% CI: 5.5–1.2), while the FCV genome was detected only in the lung (5.4%, 95% CI: 2.4–8.3) and in the intestine (8.3%, 95% CI: 0. 0–19.3). FHV-1 DNA, on the other hand, was found in brain, lung, liver, and intestine samples at percentages of 8.9% (95% CI: 0.57–17.2), 10.3% (95% CI: 6.3–14.1), 3.85% (95% CI: 0.0–11.2), and 5.5% (95% CI: 0.0–10.4), respectively.

## 4. Discussion

In this three-year study, we investigated the prevalence of some of the most important viral pathogens in cats of the Campania region. This is, to our knowledge, the first study investigating single molecular presence, risk factors (according to selected variables like year, season, location, and lifestyle), and co-infection of six viruses in cats. The overall molecular prevalence indicated that more than two-thirds of the cats (70.4%, 231/328; 95% CI: 65.5–75.4) were infected by at least one virus. When looking at the single prevalence, the most common pathogen found was Feline Panleukopenia Virus (FPV), with 73.5% of the cats tested for this virus (189/257) being positive for the virus, followed by FeLV (23.6%), FCoV (21.1%), RVA (11.4%), FHV-1 (9.05%), and FCV (7.0%).

Feline parvovirus (FPV) is clinically significant in cats and causes a high mortality rate. Here, FPV positivity in the Campania region from 2019 to 2021 was 73.5% (95% (FPV) CI: 68.2–78.9), higher than that (45.7%) detected in stray cats in a serological survey in Milan in northern Italy [10]. Nevertheless, little is known about the molecular prevalence of FPV in cats in other regions of Italy. The molecular prevalence values reported in our study were also higher than in other studies conducted in Portugal, Egypt, Turkey, and China, where the prevalences found were 58%, 45.5%, 9.7%, and 19.2%, respectively [30,31,32,33]. A possible explanation for the high percentage of positive FPV samples we found could be related, in part, to false-positive results associated with the administration of modified live vaccine within the previous 2 weeks, as also suggested by Paris et al. 2014 [34] in a study conducted in the United Kingdom. The same authors also hypothesized that the false positivity results were related to PCR cross-reaction with canine parvovirus (CPV), which is not the case of our study because we used a protocol able to distinguish between FPV and CPV [28]. 

FeLV infections cause severe disease in cats by damaging the immune system and causing malignant tumors. The virus is transmitted both horizontally and vertically, so the incidence of the disease may increase significantly in the future [35]. This infection is common worldwide, but the prevalence of the disease greatly varies between countries and regions and depends on the population studied and the diagnostic test used [36]. In our study we found a FelV genome prevalence of 23.6%, which is much higher than that described in a European study by Studer et al. (2019) [37], which found a prevalence of 1/3 or less in Italy and Malta (5.7%), Portugal (8.8%), and Hungary (5.9%). This discrepancy could be due in part to the fact that the authors tested for viral RNA while we tested for proviral DNA, and we could therefore have also detected regressive infections. Another study, also conducted in Hungary, described a molecular prevalence value of 17.3% [38], which is always lower than that revealed in our study. Lower prevalence values than those highlighted by us were also found in Ireland, China, Asia, and Taiwan, with molecular positivity rates of 11.6%, 1.5%, and 5.2%, respectively [32,39,40]. Only in Turkey was FeLV detected with a much higher prevalence (69.7%) than in our study [35]. 

FCoV is a virus with high prevalence that circulates worldwide. Infection is particularly common in environments with large numbers of cats [41]. According to Pedersen (1995) [42], the virus is present in 75–100% of domestic cats living in environments with many cats. In our study, an overall prevalence of 20.08% was found in the 266 cats examined (95% CI 16.2–25.9). Similar studies conducted in Europe have shown different prevalence values for FCoV depending on the cat population studied, but still higher than those we found in the Campania region. In Germany, 76.5% (137/179) of cats tested in breeding farms were positive for FCoV RNA in feces [43], while the authors of a study conducted in Germany found an overall FCoV prevalence of about 30.0% [44]. Higher incidence values were also found in a study conducted in Malaysia in cats from two catteries (prevalence value 84,0%) [45] and in other studies carried out in California, Florida, and Canada, where the reported prevalences were 33.0%, 47.0%, and 46.5%, respectively [46,47,48]. In Italy, Spada et al. (2016) [49] reported positivity rates of 39.0% for FCoV in a 2016 serologic survey; however, in this case, comparison of results should be made with caution due to differences in diagnostic tests. 

RVA infections rarely cause severe disease in cats and are often not properly diagnosed. However, animal rotaviruses have the potential to reassort with human strains [50]; In addition, there is strong evidence of the zoonotic potential of feline RVA strains, so RVA infections in cats must be given due consideration according to the extensive contact between humans and cats [16]. Our study, one of the first molecular prevalence research investigating the presence of RVA in cats in the Campania region and in Italy, revealed a prevalence of 11.4% (16/140, 95% CI 6.16–16.7), which is higher than that observed in a study of a cat population in the United Kingdom (3.0%, 57/1.72) and in other work performed in the past using different diagnostic techniques [17].

Respiratory infectious diseases in cats are a common clinical problem and are mainly caused by FHV-1 and FCV [51,52]. The above viruses are equally important in the development of upper respiratory tract disease in cats, although FCV seems to be the most common pathogen [53,54]. In both viral infections, cats can become virus carriers after clinical recovery: in particular, FCV carriers excrete the virus continuously, whereas FHV carriers excrete it intermittently, with the virus being latent most of the time. Widely used vaccines protect well against the disease but do not prevent infection or developmental status of the carrier [55]. In our study, the prevalence of FHV-1 (9%; 95% CI 5.36–12.74) was low and similar to prevalence values described in Switzerland (8–9%) and Turkey (12.9%) [33,56], while lower values were found in Spain (2.6%) [57]. In contrast, significantly higher prevalence values of 61.3%, 16.3%, and 17.3% were found instead in France, China, and Australia, respectively [32,58,59]. FCV, which typically leads to upper respiratory tract disease or ulcerative oral lesions [60], was detected in 7.0% (95% CI: 3.61–10.5) of the samples tested. Higher prevalence values were reported in similar studies from Switzerland (46.3%), Spain (15.5%), China (14.2%), and Australia (13.7%) [32,56,58,59].

Interestingly, when looking at the distribution of the viral genomes in the different organs investigated, wefound the presence of FPV and FHV-1 in the brain (the only two viral genomic sequences identified in this organ). Since Hora et al. (2013) [61] have already reported the involvement of FHV-1 in a case of cat severe meningoencephalitis, our results seem to confirm the ability of FHV-1 to infect cat brain [62]. As for FPV, it is known that the virus has a predominant tropism for highly mitotically active tissues [63,64], accordingly, we identified it mainly it in the spleen (see Table 9). However, in agreement with our results, the virus has also been detected in cerebral neurons of young and adult cats [64].

Overall, our results show that some of the viruses analyzed (FPV, FeLV, and RVA) seem to infect cats in our region more frequently than in other areas of the world, while FCoV and FCV seem to infect cats from our ter-rotter territories less frequently compared to other studies. However, the data shown in our work must be considered in the context of several variables, one of which is the assay used in the study. Real-Time PCR is more sensitive than other techniques [65,66,67] and may partially explain the higher prevalence we found for some of the viruses investigated. Other variables that may affect the analysis of the results must also be considered. To this end, our study analyzed the correlation between observed positivity and certain variables such as year, season, location, and lifestyle. We found a positive correlation between presence of FPV infection, year of death, and season. Indeed, we observed an OR of 6.2 and 1.82 in 2021 compared to 2019 and 2020, respectively, and in terms of seasonality, winter was considered a risk factor for infection compared to other seasons (Table 4). A positive correlation was observed between occurrence of FeLV, year of death and location, namely 2019, and Naples province showed an OR of 0.46 and 4.6, respectively, compared to the reference categories (Table 4). Contrary to what has been reported in other studies [32,38,68], we found no statistical significance between season, lifestyle, and FeLV infection, although higher prevalence values were found in summer and in cat homes (Table 4). The prevalence of FCoV, RVA, FHV-1, and FCV was higher in 2019 than in 2020 and 2021, but this figure may have been strongly influenced by pandemic-related curfews that hindered veterinary visits and laboratory testing. 

Because we were in full pandemic mode when we conducted our study, we also tested owners’ cats for the likely presence of SARS-CoV-2 genome. None of the animals tested resulted positive to the virus. As a matter of fact, the duration of SARS-CoV-2 infection and the profiles of virus shedding widely varied among the cats in the studies performed. A predisposing factor seems to be cohabitation with positive humans. Indeed, infection in cats is still considered an anthropozoonosis because it is a non-target species. Despite several positive molecular biological findings in cats tested for SARS-CoV-2, the international bibliography lacks presence compared to the numerous seroassays performed. This is due to the limited time for swabbing of a few days after exposure and during clinical manifestation of the disease. In summary, cats often do not develop clinical disease or, if they do, show only mild clinical signs that are usually respiratory or enteric in nature. These factors, together with low viral shedding, contribute to the difficulty in finding viral RNA [37,69].

## 5. Conclusions

This is the first study carried out to investigate the presence of six viral pathogens genomes in cats in Italy. In addition, molecular tests were performed to diagnose SARS-CoV-2, which showed that this virus was not present in the cat samples studied. Our results showed that viral sequences of at least two viruses were present in 20.43% of the animals, with FPV and FCoV being the most common co-infecting viruses. FPV was the most prevalent pathogen found, followed by FeLV, FCoV, RVA, FHV-1, and FCV. Cats, along with dogs, are the domestic animals most likely able to transmit viruses to humans because they live in close contact with them [49]. Therefore, surveillance of the main viral diseases affecting these animals is of great importance in order to develop optimal strategies to prevent and manage their spread, with the ultimate goal of ensuring and protecting human health.

## Figures and Tables

**Table 1:** Primers and probes employed in the Real-Time PCR assays.

Virus Investigated	Primers and Probes	Sequences	Reference
FCV	FCV-for	5′-GTAAAAGAAATTTGAGACAAT-3′	Abd-Eldaim et al., 2009 [24]
	FCV-rev	5′-TACTGAAGWTCGCGYCT-3′	
	FCV-probe	FAM-CAAACTCTGAGCTTCGTGCTTAAA TAMRA	
FCOV	FCoV-for	5′-AGCAACTACTGCCACRGGAT-3′	Dye et al., 2008 [25]
	FCoV-rev	5′-GGAAGGTTCATCTCCCCAGT-3′	
	FCoV-probe	FAM-AATGGCCACACAGGGACAACGC-MGB-3′	
FHV-1	FHV-1-for	5′-GGACAGCATAAAAGCGATTG-3′	Helps et al., 2003 [26]
	FHV-1-rev	5′-AACGTGAACAACGACGCAG-3′	
	FHV-1-probe	FAM-5′-AATTCCAGCCCGGAGCCTCAAT-MGB-3′	
FeLV	FeLV-for	5′-TCCCCAGTTGACCAGAGTTC-3′	Pinches et al., 2007 [27]
	FeLV-rev	5′-GATGGCTCGTTTTATAGCAGAAAG-3′	
	FeLV-probe	FAM-AATCCCCATGCCTCTCGCTTCTGTA-MGB-3′	
FPV	FPLV/CPV-for	5′-ACAAGATAAAAGACGTGGTGTAACTCAAATGGGAAATACAGACTATAT-3′	Decaro et al., 2008 [28]
	FPLV/CPV-rev	5′-CAACCTCAGCTGGTCTCATAATAGT-3′	
	CPV-probe	FAM-5′-ATGGGAAATACAAACTATAT-MGB-3′	
	FPV-probe	VIC-5’-ATGGGAAATACAGACTATAT-MGB-3′	
RVA	RV-JVK-for	5′-CAGTGGTTGATGCTCAAGATGGA-3′	Jothikumar et al., 2009 [29]
	RV-JVK-rev	5′-TCATTGTAATCATATTGAATACCCA-3′	
	RV-JVK -probe	5′-FAM-ACAACTGCAGCTTCAAAAGAAGWGT-3′(BHQ)	

**Table 2:** Viral genomes simultaneously present in the same animal.

Number of Viral Genomes	Virus	N. Co-Infected Cats (67 in Total)
2	FPV + FCoV	27
	FPV + FHV-1	8
	FPV + RVA	7
	FCoV + FeLV	6
	FPV + FCV	5
	FPV + FeLV	1
	FHV-1 + RVA	1
	FHV-1 + FCV	1
	FCV + FCoV	1
3	FPV + FCV + FHV-1	1
	FPV + FCV + FCoV	1
	FPV + FCoV + FeLV	1
	FCV + FCoV + RVA	1
	FCV + FHV-1 + RVA	1
	FCV + FHV-1 + FCoV	1
	FPV + FHV-1 + RVA	1
4	FPV + FCV + FCoV + FHV-1	2
	FPV + FHV-1 + FCoV + RVA	1

**Table 3:** FPV prevalence of infection and risk factor analysis in cats from Campania Region (2019–2021).

Factor	*n*	Positive	%	SE%	95% CI	X^2^	*p*	OR	95% CI
Total	257	189	73.5	5.39	68.2 78.9				
Year									
2021	109	93	85.3	4.28	90.2–98.8			Ref.	
2020	88	67	76.1	8.91	67.2–85.0	27.67	<0.0001	1.82	0.88–3.75
2019	60	29	48.3	12.6	36.7–61.0			6.2	2.89–16.9
Season									
Winter	45	43	95.5	6.0	89.5–100.0			Ref.	
Spring	52	37	71.2	12.3	58.8–83.5			10.05	2.16–46.7
Summer	76	50	65.8	10.7	55.1–76.5	14.178	0.0027	12.57	2.82–55.8
Autumn	84	59	70.2	9.8	60.5–80.0			9.48	2.13 –42.18
Location									
Napoli	129	102	79.1	7.02	72.1–87.0			Ref.	
Avellino	50	34	68.0	12.9	55.1–80.9			1.78	0.85–3.68
Benevento	34	23	67.6	15.7	51.9–83.4	6.27	0.1798	1.8	0.78–4.16
Caserta	34	25	73.5	14.8	58.7–88.4			1.36	0.56–3.25
Salerno	10	5	50.0	31.0	19.0–81.0			3.77	1.01–14.0
Lifestyle									
Outdoor	116	89	76.7	7.69	69.0–84.4			Ref.	
Indoor	58	41	70.7	11.7	58.9–82.4	1.104	0.57	1.36	0.67–2.78
Cat shelter	83	59	71.1	9.75	61.3–80.8			1.34	0.70–2.54

**Table 4:** FeLV infection prevalence and risk factor analysis in cats from Campania Region (2019–2021).

Factor	*n*	Positive	%	SE%	95% CI	X^2^	*p*	OR	95% CI
Total	89	21	23.6	8.82	14.7–32.4				
Year									
2021	32	6	18.7	13.5	5.23–32.3			Ref.	
2020	15	1	6.67	12.6	0.00–19.3	9.165	0.0102	3.23	0.35–29.5
2019	42	14	33.3	14.3	19.08–47.6			0.46	0.15–1.37
Season									
Winter	7	2	28.5	33.5	0.00–62.06			Ref.	
Spring	19	2	10.5	13.8	0.00–24.3	5.153	0.1609	3.4	0.37–30.6
Summer	53	17	32.1	12.6	19.5–44.6			0.84	0.14–4.81
Autumn	10	0	-	-	-			-	-
Location									
Napoli	40	15	37.5	15.0	22.5–52.5			Ref.	
Avellino	26	3	11.5	12.3	0.00–23.8			4.6	1.17–17.9
Benevento	20	3	15.0	15.6	0.00–30.6	9.783	0.044	3.4	0.85–13.5
Caserta	1	0	-	-	-			-	-
Salerno	2	0	-	-	-			-	-
Lifestyle									
Outdoor	30	4	13.3	12.2	1.17–25.5			Ref.	
Indoor	9	1	11.1	20.5	0.00–31.6	4.49	0.105	0.88	0.08–9.76
Cat shelter	50	16	32.0	12.9	19.07–44.9			0.21	0.05–0.81

**Table 5:** FCoV prevalence of infection and risk factor analysis in cats from Campania Region, in the period 2019–2021.

Factor	*n*	Positive	%	SE%	95% CI	X^2^	*p*	OR	95% CI
Total	266	56	21.1	4.9	16.2–25.9				
Year									
2021	109	21	19.3	7.4	11.9–26.7			Ref.	
2020	81	9	11.1	6.8	4.3–17.9	12.94	0.0015	1.9	0.82–4.25
2019	76	26	34.2	10.7	23.5–44.8			0.458	0.23–0.89
Season									
Winter	40	12	30.0	14.2	15.8–44.2			Ref.	
Spring	45	10	22.2	12.2	10.1–34.4			1.5	0.56–3.97
Summer	103	24	23.3	8.16	15.1–31.5	1.109	0.774	1.41	0.62–3.19
Autumn	78	10	12.8	7.42	5.4–20.2			2.91	1.12–7.51
Location									
Napoli	130	35	26.9	7.62	19.3–34.5			Ref.	
Avellino	61	11	18.03	9.65	8.4–27.7			1.67	0.78–3.57
Benevento	33	5	15.2	12.2	2.92–27.4	6.066	0.194	2.06	0.73–5.76
Caserta	30	3	10.0	10.7	0.0–20.7			3.31	0.94–11.6
Salerno	12	2	16.7	21.09	0.0–37.7			1.84	0.38–8.82
Lifestyle									
Outdoor	111	20	18.02	7.15	10.9–25.2			Ref.	
Indoor	54	17	31.5	12.4	19.1–43.9	4.454	0.128	0.47	0.22–1.01
Cat shelter	101	19	18.8	7.62	11.2–26.4			0.94	0.47–1.9

**Table 6:** RVA prevalence and risk factor analysis in cats from Campania Region (2019–2021).

Factor	*n*	Positive	%	SE%	95% CI	X^2^	*p*	OR	95% CI
Total	140	16	11.43	2.69	6.16–16.7				
Year									
2021	68	5	7.35	6.2	1.15–13.6			Ref.	
2020	41	1	2.5	4.84	0.00–7.34	17.676	0.0001	3.17	0.35–28.2
2019	31	10	32.3	16.5	15.8–48.7			0.16	0.05–0.54
Season									
Winter	24	4	16.7	14.9	1.76–31.6			Ref.	
Spring	22	1	4.5	8.7	0.00–13.2			4.2	0.43–41.0
Summer	44	6	13.6	10.1	3.5–23.8	1.993	0.5739	1.3	0.32–5.01
Autumn	50	5	10.0	8.3	1.7–18.3			1.8	0.43–7.41
Location									
Napoli	69	8	11.6	7.5	4.04–19.15			Ref.	
Avellino	29	1	3.4	6.6	0.00–10.1			3.67	0.43–30.8
Benevento	6	0	-	-	-	4.977	0.2896	-	-
Caserta	27	5	18.5	14.6	3.87–33.2			0.57	0.17–1.95
Salerno	9	2	22.2	27.2	0.00–49.4			0.45	0.08–2.6
Lifestyle									
Outdoor	72	9	12.5	7.64	4.86–20.14			Ref.	
Indoor	32	4	9.52	8.88	0.65–18.4	0.459	0.7951	1	0.28–3.52
Cat shelter	36	3	8.33	9.03	0.00–17.4			1.57	0.39–6.2

**Table 7:** FHV-1 prevalence of infection and risk factor analysis (Campania Region, 2019–2022).

Factor	*n*	Positive	%	SE%	95% CI	X^2^	*p*	OR	95% CI
Total	232	21	9.05	3.69	5.36–12.74				
Year									
2021	96	4	4.17	4.0	0.17–8.16			Ref.	
2020	83	8	9.64	6.35	3.3–15.9	6.86	0.0323	0.40	0.12–1.4
2019	53	9	16.9	10.1	6.87–27.09			0.21	0.06–0.72
Season									
Winter	46	4	8.7	8.14	0.55–16.8			Ref.	
Spring	42	2	4.76	6.64	0.00–11.2			1.9	0.33–10.9
Summer	73	6	8.22	6.3	1.92–14.5	1.260	0.738	1.06	0.28–3.99
Autumn	64	7	10.9	7.6	3.3–18.6			0.76	0.20–2.77
Location									
Napoli	120	14	11.6	5.74	5.9–17.4			Ref.	
Avellino	46	1	2.17	4.21	0.00–6.39			5.9	0.75–45.5
Benevento	30	2	6.67	8.93	0.00–15.59	30.362	<0.0001	1.8	0.39–8.61
Caserta	30	4	13.3	12.2	1.2–25.5			0.85	0.26–2.82
Salerno	6	0	-	-	-				
Lifestyle									
Outdoor	96	11	11.5	6.4	5.1–17.8			Ref.	
Indoor	61	4	6.5	6.2	0.35–12.7	1.237	0.5387	1.84	0.55–6.07
Cat shelter	75	6	8.0	6.14	1.9–14.1			1.5	0.52–4.22

**Table 8:** Prevalence of infection of FCV and risk factor analysis (Campania Region, 2019–2021).

Factor	*n*	Positive	%	SE%	95% CI	X^2^	*p*	OR	95% CI
Total	213	15	7.04	1.75	3.61–10.5				
Year									
2021	83	1	1.2	2.35	0.00–3.55			Ref.	
2020	81	11	13.6	7.46	6.12–21.0	8.957	0.0113	0.084	0.01–0.67
2019	49	4	8.16	7.67	0.50–15.8			0.13	0.01–1.24
Season									
Winter	48	3	6.3	6.9	0.00–13.1			Ref.	
Spring	37	1	2.7	5.2	0.00–7.93			1.56	0.13–17.9
Summer	61	5	8.2	6.9	1.31–15.1	1.275	0.7351	0.63	0.11–3.6
Autumn	67	6	8.9	6.8	2.12–15.8			0.54	0.1–2.94
Location									
Napoli	106	10	9.43	5.56	3.87–15.0			Ref.	
Avellino	45	1	2.22	4.31	0.00–6.53			4.5	0.56–36.9
Benevento	28	1	3.57	6.87	0.00–10.5	9.889	0.0423	2.8	0.34–22.9
Caserta	28	1	3.57	6.87	0.00–10.5			2.8	0.34–22.9
Salerno	6	2	33.3	37.7	0.00–71.1			0.2	0.03–1.3
Lifestyle									
Outdoor	85	4	4.71	4.5	0.2–9.2			Ref.	
Indoor	56	2	3.57	4.8	0.00–8.43	0.408	0.815	1.3	0.2–7.5
Cat shelter	72	9	12.5	7.6	4.86–20.1			0.34	0.1–1.2

**Table 9:** Percentage of positive organs with respect to the virus investigated. FPV DNA was prevalently found in the spleen (72.5%), FelV and FCov genomes in the heart (41.4% and 28.9%, respectively), RVA and FCV nucleic acids in the intestine (10.4% and 8.3%, respectively); FHV-1 DNA in the lungs (10.3%). The highest prevalence/virus genomes are in bold.

Organ	Virus Investigated					
	FPV	FeLV	FCoV	RVA	FHV-1	FCV
Brain	61.4%				8.9%	
	(CI 95% 46.9–75.7)	-	-	-	(CI 95% 0.57–17.2)	-
Heart	70.8%	**41.4**%	**28.9**%			
	(CI 95% 62.1–79.6)	(CI 95% 27.4–60.8)	(CI 95% 14.5–43.3)	-	-	-
Lung	68.2%	11.1%	8.8%		**10.3**%	5.4%
	(CI 95% 61.6–74.7)	(CI 95% 0.8–21.3)	(CI 95% 1.9–20.7)	-	(CI 95% 6.3–14.1)	(CI 95% 2.4–8.3)
Liver	70.2%	11.5%	16.3%	3.8%	3.8%	
	(CI 95% 62.4–78.1)	(CI 95% 0.0–23.8)	(CI 95% 3.2–29.1)	(CI 95% 0.0–11.2)	(CI 95% 0.0–11.2)	-
Spleen	**72.5%**		15.8%			
	(CI 95% 62.9–81.1)	-	(CI 95% 0.0–32.2)	-	-	-
Intestine	71.5%	20.0%	18.1%	**10.4%**	5.6%	**8.3%**
	(CI 95% 65.7–77.2)	(CI 95% 4.3–35.6)	(CI 95% 13.1–23.1)	(CI 95% 5.5–1.2)	(CI 95% 0.0–10.4)	(CI 95% 0. 0–19.3)
